# Frailty, Quality of Life, Anxiety, and Other Factors Affecting Adherence to Physical Activity Recommendations by Hemodialysis Patients

**DOI:** 10.3390/ijerph16101827

**Published:** 2019-05-23

**Authors:** Beata Hornik, Jan Duława

**Affiliations:** 1 Department of Internal Nursing, School of Health Sciences in Katowice, Medical University of Silesia, 40-752 Katowice, Poland; 2 Department of Internal Medicine and Metabolic Diseases, School of Health Sciences in Katowice, Medical University of Silesia, 40-752 Katowice, Poland; jdulawa@sum.edu.pl

**Keywords:** hemodialysis, frailty, adherence, physical activity, anxiety, quality of life, acceptance of illness, functional status, motivation

## Abstract

Hemodialysis patients perform little physical activity. We formulated a hypothesis that some factors, i.e., frailty, medical and functional factors, psychological factors, quality of life, awareness of recommendations, and sociodemographic factors influence the decisions of taking up physical activity. This prospective study comprised 72 dialysis patients aged 57.8 ± 16.0 ($\bar{x}$ ± SD; in the range of 19–87 years of age). The following research tools were used: an interview about awareness of the physical activity recommendations, the Canadian Study of Health and Aging Scale (CSHA-CFS), scales for the assessment of functional status, State-Trait Anxiety Inventory (STAI), Acceptance of Illness Scale (AIS), and the questionnaire of Kidney Disease Quality of Life (KDQOL-SF 1.3). The majority of patients diagnosed with frailty did not follow the physical activity recommendations (79.3%). Quality of life was better in active patients compared to inactive patients, especially in the domains of sleep and physical performance. The severity of trait anxiety was significantly higher in patients who did not follow the recommendations compared to patients who adhered to physical activity recommendations (46.0 ± 10.5 vs. 40.0 ± 8.2; p=0.021). The likelihood of adherence decreased by 1% after each subsequent month of dialysis (odds ratio = 0.99; 95% confidence interval = 0.972–0.999; p=0.047). Adherence was most limited by frailty. Adherence to recommendations on physical activity was affected by: motivation, lower levels of trait anxiety, and better quality of life. Age modified the effect of awareness and acceptance of the disease on adherence to physical activity recommendations.

## 1. Introduction

Patients undergoing chronic dialysis are at particularly high risk of reduced physical activity. Lower exercise capacity causes decreased quality of life, higher morbidity and mortality, and decreased functional fitness [1,2,3,4,5,6,7]. Nowadays, international guidelines recommend that chronic kidney disease patients should engage in an exercise program that is compatible with cardiovascular tolerance for at least 30 min, 5 days of the week [8]. Physical fitness in dialysis patients is only 50–60% of the fitness in the population of their sedentary contemporaries [9]. Some reports suggest that such patients spend only 25 min a week on walking or other light physical activity. Merely 17% of them take some form of moderate physical activity that lasts 150 min per week [10]. Physical activities limited to less than 4000 steps a day increase mortality risk in hemodialysis patients [11].

The beginning of hemodialysis, especially in older patients, is usually associated with a significant and progressive deterioration of functionality, particularly in the first year of treatment [12]. It is not known to what degree the decline of functional fitness is the result of the end-stage renal disease and dialysis therapy, and to what degree it is the effect of limited physical activity.

Even in very old individuals with serious medical issues, physical activity improves functional fitness [9,13,14,15]. In end-stage renal disease, uremia symptoms are often accompanied by frailty. These symptoms are linked to biological aging and occur at an earlier age in chronic dialysis patients than in the general population, which strongly correlates with prognosis [16,17]. For this reason, ESRD can be treated as a model of accelerated aging. There are many similarities between uremia stages and aging, so the obtained results from hemodialysis patients can be extrapolated to the population of older individuals [18]. Frailty occurred in 24–75% of dialysis patients, depending on the evaluation method; diagnosis of frailty was independent of age [19,20,21,22,23,24].

Risk factors for developing frailty include, among other things, low physical activity. Studies on adherence to the recommended physical activity carried out in the USA [10], China [25], and Italy [26] revealed that the factors contributing to low physical activity in hemodialysis patients are linked to socioeconomic, medical, psychological, and cultural barriers, as well as the organization of medical care and dialysis personnel [27,28,29,30,31]. Only a few studies consider the combined effect of multiple factors, i.e., awareness of recommendations, sociodemographic, medical, and functional factors, including frailty, psychological factors, and quality of life.

The objective of this study was to assess the level of adherence to recommended physical activity among hemodialysis patients and to identify factors and limitations that have the greatest influence on the decisions that hemodialysis patients make to take up physical activity.

## 2. Materials and Methods

This prospective study comprised 72 dialysis patients (36 women and 36 men) aged 57.8 ± 16.0 ($\bar{x}$ ± SD; ranging 19–87 years of age). Patients had been dialyzed for an average of 46.2 ± 41.4 months (2–269 months) and met the inclusion criteria. All were treated at the same dialysis station. Following a detailed explanation of the study purpose and protocol, each patient submitted their written consent.

The inclusion criteria were as follows: >18 years old, end-stage renal disease and hemodialysis treatment, ability to answer open questionnaire questions, and the patients’ informed and voluntary consent. The exclusion criteria included: disease or consciousness disorders hindering the patient’s capacity for participation in an interview or completing a questionnaire (New York Heart Association Class IV heart failure, confusions, impaired consciousness), mental illness, advanced cancer, and lack of consent to participate in the study.

The study was approved by the Bioethical Committee of the Medical University of Silesia in Katowice (Resolution No. KNW/0022/KB1/44/II/06/14/17) and was carried out in accordance with the Declaration of Helsinki regarding human studies.

### 2.1. Methods

All patients underwent routine laboratory tests and basic physical tests. Fasting blood samples (at least 12 h after the last meal) were taken. Laboratory tests included blood hemoglobin concentration, leucocytes count, as well as creatinine, sodium, and potassium serum concentrations. Serum urea concentrations were measured before and after dialysis. Natural logarithm of the ratio of urea concentrations before and after dialysis (Kt/V) was used to quantify the adequacy of dialysis. The following measurements were taken: body weight, body mass index (BMI), and waist circumference. Additionally, subjects underwent geriatric functional assessment. The evaluation focused on the level of adherence to the recommended regular physical activity. The impact of recommendation awareness, and medical and functional factors including frailty, psychological factors, and quality of life was also assessed.

### 2.2. Awareness of Recommendations

Before assessing the awareness of physical activity recommendations, each patient who qualified for the repeated dialysis treatment received the necessary information and education materials on the course of treatment and basic recommendations including exercise. The information was presented by a doctor and nephrology nurse. Awareness of recommendations was assessed on a three-point scale: patient remembers without being reminded, patient remembers after being reminded, or patient does not remember. The categories: patient remembers without being reminded and patient remembers after being reminded were combined into a single category in the final analysis: patient remembers.

Patients whose knowledge of recommendations was confirmed as consistent with the National Kidney Foundation (K/DOQI) [32,33] were considered aware of recommendations. Adherence to the recommended physical activity was evaluated using an indirect method based on the patient’s declared behavior during a two-week period prior to the study. Patients who engaged in moderate physical activity (at least 90–150 min per week) or light physical activity (at least 30 min 5 times per week) were considered adhering to physical activity recommendations.

### 2.3. Medical Factors

The assessment included vascular access type and the cause of end-stage renal disease. The Charlson Comorbidity Index (CCI) was determined for each patient. For every decade above 40 years of age, one point was added to the score [34]. Occurrence of frailty was assessed using the Canadian Study of Health and Aging Scale (CSHA-CFS), a 7-step scale that allows obtaining a global clinical measurement of biological age, disabilities, and cognitive impairment. Frailty was identified on the basis of scoring 5 or more points on the CSHA-CFS scale. Patients who scored 4 points were at risk of pre-frailty, while scores ranging 1–3 points (robust) meant that frailty was not detected [35].

### 2.4. Scales for Assessment of Functional Status

#### 2.4.1. Scale for Assessing Activities of Daily Living (ADL), Katz Index

The index included 6 questions about the level of performance in basic daily activities (bathing, dressing, toileting, transferring, continence and feeding). Every activity was evaluated on a 2-point scale. Six points were the maximum score (5–6 points indicate a norm, 3–4 points indicate moderate impairment, and 0–2 points indicate severe impairment) [36].

#### 2.4.2. Scale for Assessing Instrumental ADL (IADL), Lawton Index

The scale assesses nine instrumental activities (using the telephone, moving within the community, shopping, preparing meals, cleaning the house, doing laundry, using public transport, taking prescribed medications, and managing money). A 3-point scale was used to evaluate each parameter. The highest possible score that could be achieved was 27 points. Higher scores indicate greater ability to perform these activities [37].

#### 2.4.3. Barthel Index (Barthel Index of Activities of Daily Living)

The index is a commonly used tool to assess the need for third-party care. It uses points to assess daily activities: feeding, mobility, bathing, etc. The scores are in the range of 0–20 points. Higher scores indicate better functioning: 0–4 points indicate total dependency, 5–9 points indicate medium dependence, 10–14 points indicate partial dependence, 15–19 points indicate moderate dependence, and 20 points indicate independence [38].

#### 2.4.4. Mobility-Independence Scale

The scale assesses a patient’s independence related to the actual range of independent mobility (one can use any orthopedic equipment but without another person’s assistance). The results ranged 0–9 (0—bedridden, 1—unassisted change of position in bed, 2—sitting up, 3—standing up or a wheelchair near the bed, 4—moving a few meters around the room, 5—ability to walk on flat surfaces inside and around the house, 6—walking on flat surfaces within the neighborhood, 7—walking on uneven surfaces and climbing stairs inside the house, 8—walking on uneven surfaces and climbing stairs outside, and 9—walking on uneven surfaces and climbing stairs without restrictions). A higher score indicates a greater ability to move independently [39,40].

#### 2.4.5. Scales for Functional Assessment of Hearing and Sight

The scales assess the state of the senses related to the ability to carry out daily activities and engage in interpersonal communication; if the patient uses eyeglasses or a hearing aid, they are included in the evaluation. Patients self-asses their eyesight and hearing. The results are in the range of 0–4 (0—no activity, 1—significant limitation, 2—moderate limitation, 3—minor limitation, 4—normal hearing and right or full deficit correction) [41].

### 2.5. Psychological Factors

#### 2.5.1. State-Trait Anxiety Inventory (STAI)

STAI tests two types of anxiety: X1—state anxiety, and X2—trait anxiety. State anxiety is a complex emotional reaction that consists of a subjective, nonspecific feeling of tension and threat. Trait anxiety has been defined as an individual and more permanent disposition to reacting with fear and perceiving a given situation as threatening. The results range in 20–80 raw points and can be transformed into ranges (stens). This study uses raw results [42].

#### 2.5.2. Acceptance of Illness Scale (AIS)

The scale was developed by Felton, Revenson, and Hinrichsen [43] and was adapted to Polish requirements by Juczyński [44]. The scale contains 8 statements describing limitations caused by the disease. A decidedly affirmative answer—1 indicates poor adaptation to the illness and negative response, while 5 shows the patient has come to terms with the disease. The sum of points (8–40) indicates the degree of acceptance. The scale shows a positive trend.

### 2.6. Motivation

The motivation-assessment scale was created for the needs of this study and is based on the motivation scale for diabetes patients [45]. It consists of 14 testing items that use a Likert-style measuring scale (from 1—never or almost never to 5—very high degree) to evaluate the level of motivation to follow medical recommendations. The highest motivation is represented alternately by 5 (direct statements) or by 1 (indirect statements). Reversed scoring was used in the key next to the indirect statements. Patients score 14–70 points. A greater number of scored points suggests stronger motivation.

### 2.7. Quality of Life

#### Kidney Disease Quality of Life (KDQOL-SF 1.3)

The questionnaire determines quality of life in 4 categories (physical, psychological, interpersonal, and environmental), including specific problems encountered by patients with chronic kidney disease. There are 36 questions about general health and 43 questions about kidney disease. Raw data from each question were converted and presented on a 0–100 scale. Higher scores indicate better quality of life. Questions were grouped into subscales (domains). Questions about general health were arranged into 8 domains, and questions about kidney disease were organized into 11 domains [46].

### 2.8. Statistical Analysis

Continuous variables were expressed as mean values ± standard deviations (SD) and categorical variables as counts and percentages. For the majority of analyses, a comparison of parametric values between the two groups was made using Student’s *t*-test and Mann–Whitney *U* test. The Mann–Whitney *U* test was used to compare two non-normally distributed variables, while the Student’s *t*-test was used for the comparison of normally distributed continuous variables.

The relationships between variables estimated using weak measuring scales were identified by the likelihood-ratio test. Correlation analysis was used to examine the relationship, and Cramér’s V coefficient was defined. Cramér’s V coefficient ranges from 0 (no association between variables) to 1 (positive relationship between variables).

Multivariate analysis with logistic regression was carried out to investigate the impact of independent variables on the dichotomous dependent variable. Multivariate analysis with logistic regression included variables whose returned *p*-value ≤ 0.1 in the initial univariate analysis. Odds ratios (ORs) and 95% confidence intervals (CIs) were determined. The level of significance was set at *p* < 0.05. The analysis of basic statistics and correlation was performed with the use of statistics software R version 3.1.1 (R Foundation for Statistical Computing, Vienna, Austria). R is an open source project [47]. Regression analysis was performed using Statistica 13.1 (StatSoft, Inc., Tulsa, OK, USA).

## 3. Results

Patient characteristics are shown in Table 1. The major causes of end-stage renal failure are: diabetic renal disease (29.2%), glomerulonephritis (22.2%), hypertensive nephropathy (16.7%), and other conditions, e.g., systemic diseases, polycystic kidney disease, and urological conditions (31.9%).

Vascular access was through an arteriovenous (AV) fistula in 41 patients (56.9%), through AV graft in 8 patients (11.1%), and dialysis catheter in 23 patients (32%). Bicarbonate-dialysis sessions lasted for 252.8 ± 14.5 ($\bar{x}$ ± SD) min (range of 240–270 min) and were carried out 3–4-times a week using high-flux dialysis machines. Dialysis fluid-flow rate was 500 mL/min, and blood flow 250–350 mL/min. The mean comorbidity index (CCI) was 4.9 ± 2.4 (range 2–12). Frailty was diagnosed in 29 patients (40.3%), while 11 patients (15.3%) were diagnosed with pre-frailty. Half of patients (50%) failed to perform any form of physical activity (Table 1).

### 3.1. Awareness of Recommendations

Adherence to recommended physical activity was significantly more frequent in patients who were aware of such recommendations compared to those who were unaware (60% vs. 27.3%; p<0.001). There was moderate correlation (Cramér’s V 0.321) between recommendation awareness and adherence to it (Table 2).

### 3.2. Sociodemographic Factors

Significant weak correlation between gender and adherence to recommended physical activity was observed (Cramér’s V 0.278). Women were less likely to adhere to recommended physical activity than men (36.1% vs. 63.9%; p=0.018) (Table 2). Adherers to recommended physical activity were younger than non-adherers (50.8 ± 15.9 vs. 64.7 ± 13.0 years of age; p<0.001) (Table 3). Other sociodemographic factors (education, marital status) did not have any significant impact on adherence to regular physical activity (Table 2).

### 3.3. Medical Factors

**Figure 1:**
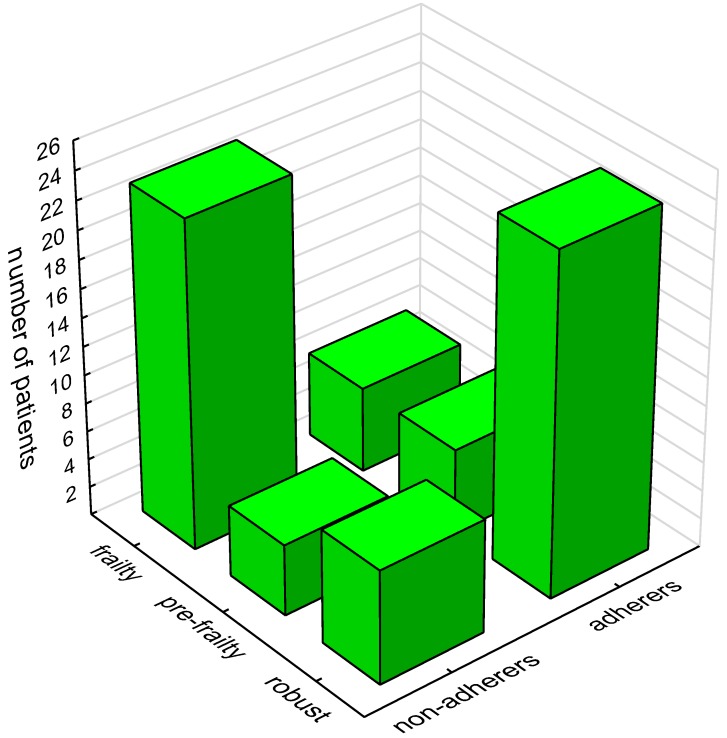
Influence of frailty on adherence to physical activity recommendations.

The majority of patients diagnosed with frailty did not follow physical activity recommendations (79.3%) (Table 2) (Figure 1). Adhering to recommended regular physical activity was significantly related to the cause of end-stage renal disease (Cramér’s V 0.363; p=0.020) and vascular access type (Cramér’s V 0.325; p=0.043). Patients with ESRD due to glomerulonephritis were found to follow physical activity recommendations significantly more often, while the presence of diabetes was associated with lower adherence to recommendations. The majority of patients with arteriovenous fistula followed physical activity recommendations more frequently compared to patients with a central catheter (63.4% vs. 34.8%) (Table 2). Physically active patients had a significantly lower comorbidity rate compared to physically inactive individuals (3.9 ± 2.0 vs. 5.9 ± 2.6; p<0.001) (Table 3).

### 3.4. Functional Status

All measurement scales revealed that patients with higher functional status adhered better to recommended physical activity as compared to those with low functional stages: ADL 5.9 ± 0.4 vs. 5.0 ± 1.9, p<0.001; IADL 23.6 ± 4.0 vs. 17.1 ± 6.6, p<0.001; and Barthel 19.6 ± 1.1 vs. 16.7 ± 3.9, p<0.001. A significant correlation between movement distances and physical activity was observed (Cramér’s V 0.622; p<0.001). Eighty-two percent of patients following physical activity recommendations were capable of traveling outside their place of residence. Patients who were only able to move around the premises of their home or apartment did not follow any form of physical activity. A large percentage (65.3%) of patients, whose visual impairment did not cause considerable difficulties with normal daily activities, adhered to the recommendations. Functional hearing loss did not significantly affect adherence to physical activity recommendations (Table 3).

### 3.5. Psychological Factors

Disease acceptance scores and motivations were significantly higher in patients who followed physical activity recommendations compared to inactive patients (23.3 ± 7.9 vs. 19.8 ± 8.3; p=0.039 and 65.9 ± 8.8 vs. 58.6 ± 8.1; p<0.001, respectively). The severity of trait anxiety was significantly higher in patients who did not follow recommendations compared to patients who did (46.0 ± 10.5 vs. 40.0 ± 8.2; p=0.021). The severity of state anxiety did not significantly differ between patients who adhered to recommendations and those who did not (Table 3).

### 3.6. Quality of Life

Sleep quality ratings were better in patients who adhered to recommendations compared to inactive patients. Quality of life was also better in active compared to inactive patients with respect to the following domains: somatic disorders (77.20 ± 13.78 vs. 70.20 ± 14.55; p=0.047), medical personnel support (72.22 ± 27.76 vs. 57.64 ± 27.67; p=0.015), and social support (80.09 ± 21.39 vs. 68.06 ± 20.89; p=0.018) (Table 4).

Individuals who followed the recommendations had a better quality of life in general domains: physical functioning (65.97 ± 29.67 vs. 32.78 ± 29.37; p<0.001), energy/fatigue (52.64 ± 22.34 vs. 36.25 ± 21.98; p=0.002), role limitations—physical (50.00 ± 43.77 vs. 28.47 ± 43.18; p=0.043), and overall physical-health assessment (39.34 ± 11.70 vs. 32.34 ± 11.57; p<0.001). However, adherers significantly differed from non-adherers in the domain of social functioning (65.28 ± 31.75 vs. 46.18 ± 31.25; p=0.010). Regarding the remaining specific and general domains, quality of life was not significantly different between patients who followed recommendations and those who did not (Table 4).

#### Regression

Three models of logistic regression were created to determine factors that account for taking up physical activity. Each model included factors significant in the initial univariate analysis. The first model was age-independent, the second for patients over 60 years of age, and the third model was used for patients under 60 years of age (Table 5).

The logistic regression age-independent model demonstrated that vascular access type and recommendation awareness had the most marked effect on adherence to physical activity recommendations (odds ratio (OR) = 0.09; 95% CI = 0.017–0.530; p=0.007 and OR = 0.08; 95% CI = 0.013–0.464; p=0.005, respectively). Frailty measured by the CSHA-CFS scale reduced chances of adherence by 62%, while motivation increased by 13% with each additional point. The likelihood of adherence decreased by 1% after each consecutive month of dialysis (Table 5).

The logistic regression model for individuals over the age of 60 incorporated five factors linked to adherence to recommended physical activity, i.e., CSHA-CFS, motivation, AIS, hemodialysis-treatment duration, and vascular access type. Adherence to recommended physical activity in patients over 60 years of age was most significantly affected by frailty, whose occurrence lowered the chances of adherence to recommendations by 75% with each consecutive point on the CSHA-CFS scale (OR = 0.25; 95% CI = 0.071–0.861; p=0.028) followed by AIS (OR = 0.79; 95% CI = 0.640–0.990; p=0.036) and the duration of hemodialysis treatment (OR = 0.95; 95% CI = 0.918–0.999; p=0.025). Patients with an arteriovenous fistula were more likely to follow recommendations than those with other vascular access (OR = 0.09; 95% CI = 0.008–0.950); p=0.045). The impact approached significance (Table 5).

In patients below 60, lack of awareness of physical activity recommendations (OR = 0.02; 95% CI = 0.001–0.427; p=0.012) and frailty measured by the CSHA-CFS scale (OR = 0.13; 95% CI = 0.026–0.633; p=0.012) had the most considerable effect on adherence. Each additional point on CSHA-CFS decreased the chances of following recommendations by 87%. Greater awareness of recommendations increased the chances for physical activity (Table 5).

## 4. Discussion

Half of our study patients declared minimal physical activity. Numerous studies confirm inactive behaviors of hemodialysis patients [48,49,50], resulting in poor physical fitness [5,6,7] and shorter survival [1]. This study aimed to analyze a number of medical, functional, sociodemographic, and psychological factors, as well as those connected to quality of life that might affect adherence to recommended physical activity.

Frailty proved to be a considerable limitation in taking up physical activity, regardless of age. Our study appears to be the first to demonstrate the effect of frailty on adherence to recommendations in hemodialysis patients, also of younger age. Depending on assessment method, several authors reported frailty was three- to tenfold more common in hemodialysis patients compared to older subjects with normal renal function [5,17,22,51,52]. Since low physical activity is a criterion in frailty definition [53], interventions leading to increased physical activity might reduce or reverse frailty status, both directly and indirectly, provided they improve physical fitness or reduce the symptoms of fatigue and exhaustion [54]. Patients’ engagement in physical activity was inversely proportional to comorbidity index, particularly in the presence of diabetes. It should be noted, however, that comorbidities such as hypertension, diabetes, cardiovascular disorders, hyperlipidemia, and degenerative changes in muscles and bones do not rule out physical exercises. In fact, patients with these conditions should be encouraged to take up physical activity [55]. Better adherence to recommendations in patients with ESRD due to glomerulonephritis compared to those in whom diabetes was the primary disease, may have been caused by overall clinical status. Diabetes is a systemic disease, and diabetic patients may experience multiorgan complications. The low physical activity of these patients might have been related to neuropathic pain associated with diabetic kidney disease [56]. Hemodialysis patients have often coexisting diabetes and one of their comorbidities is peripheral neuropathy and reduced sensation in the feet [57]. Another reason could be the correlation between frailty and diabetic nephropathy observed in studies by Kakio et al. [58]. Furthermore, low physical activity in patients with concurrent diabetes might indicate their tendency toward sedentary behaviors even before ESRD diagnosis.

The type of vascular access strongly affected engagement in physical activity. Patients with vascular catheters less frequently followed recommendations. The goal, if possible, is to create an arteriovenous fistula using a patient’s vessels. Physical exercises for patients with a vascular catheter should be adapted to this limitation. Patients with a vascular catheter tend to limit physical activity, as they fear catheter displacement, infection, local injury, and bleeding [59]. It remains undetermined thought whether patients with arteriovenous fistula would be more active. We did not observe any effects of vascular access type in younger patients, but it should be noted that none of our study participants had a catheter inserted into the femoral vein, which would have hindered physical exercise irrespective of age. Another issue is that catheter placement typically indicates poor condition of the vessels and unfavorable clinical status of the patient causing them to abstain from partaking in recommended physical activity.

In elderly patients, adherence to recommended physical activity was negatively correlated with the time spent in a chronic dialysis program. With each subsequent month of dialysis, the chances to take up physical activity decreased. This observation is consistent with previous reports [10,60]. Patients who only moved within their own house or apartment were defined as completely inactive. This was probably caused by low physical fitness, which naturally leads to a significant limitation of physical activity. Such patients should therefore be included in exercise programs aiming to prevent disability progression. It is worth noting that even slight functional vision impairment and resulting function impairment significantly reduced physical activity levels. This indicates the need for the best vision correction possible and inclusion in interventions designed to enhance physical activity. Smith et al. reached a similar conclusion after studying a large general-population sample (6634 older adults; mean age 65 ± 9.2 years). They found that study participants with vision defects were twice as likely to be inactive than individuals who rated their eyesight as excellent [61]. Other studies have shown that hemodialysis patients encounter a wide range of medical and psychological problems that make it difficult to take up physical activity [26,60,62,63,64].

The logistic regression model did not confirm the effect of awareness of physical activity recommendations on adherence by older patients (≥60 years of age). However, awareness of these recommendations was an important factor in taking up physical activity by younger patients (<60 years of age). Awareness of recommendations and adherence are closely related [65]. It should be emphasized that the importance of awareness of recommendations can only be developed as a result of patient education, an essential element of dialysis care [6,66,67,68,69].

In this study, engagement in physical activity was significantly influenced by psychological factors. The effect of adequate coping with the disease on adherence to recommendations on physical activity was ambiguous. Better coping was a predictor of increased physical activity in younger patients, while in older patients, more adequate coping was associated with decreased physical activity. The practical significance of this observation remains to be clarified. Many authors emphasize that the level of disease acceptance increases with self-esteem and self-efficacy [70]. Patients who followed physical activity recommendations were more accepting of their illness, but its importance changed in the group of factors in individuals over 60 years of age. Frailty, higher levels of disease acceptance, and each additional month of renal replacement treatment turned out to be factors that diminished chances of adherence to recommendations. It might be that frailty was the most significant limitation in taking up physical activity, and therefore it weakened the effect of adequate coping. However, this observation should be treated cautiously, since several authors observed that acceptance of the disease and treatment methods is necessary for patients to continue with therapy and comply with the doctor’s recommendations [71,72,73]. Other authors point out that disease acceptance is an independent factor positively correlated with quality of life (QoL) [74,75]. Harrison et al. demonstrated that acceptance of the disease increased with age, as various dysfunctions are more easily accepted by older individuals [76]. In this study, it was older patients who did not adhere to physical activity recommendations. This observation is consistent with the development theory that assumes that older chronically ill individuals exhibit decreased vitality [77].

Motivation was undoubtedly a factor that was significantly related to adherence to recommendations on physical activity. Motivation was of greater importance in patients aged ≥60. Other studies confirm that the effectiveness of treatment largely depends on the patient’s motivation [78,79,80]. Motivating patients to change their lifestyle is frequently much more challenging than getting them to cooperate on, for example, a pharmacotherapy regimen. Positive encouragement works better than fear of disease consequences [81,82,83]. Reinforcing a positive outlook on the future (“dispositional optimism”) may facilitate treatment. The attitude of so-called learned pessimism reduces cooperation and motivation. While dispositional optimism and learned pessimism are relatively constant traits, environmental factors are also important [84]. In this study, trait anxiety (a relatively constant personality trait) had a significant effect on physical activity; its severity was significantly lower in patients who adhered to physical activity recommendations. State anxiety was comparable in active and nonactive patients.

Other studies have shown that non-adherence might be associated with depression, anxiety, and hostility [85]. Anxiety is a factor affecting the degree of adherence to recommendations. The resultant physical activity lowers anxiety and enhances the mood [86], and therefore may at least be as effective in treating depression as other methods, including pharmacotherapy [87,88]. There is ample evidence that depressed patients frequently demonstrate behaviors inconsistent with recommendations [89,90]. The correlation between adherence to recommendations and anxiety is not as explicit as that between adherence and depression [91].

Quality of life and its relationship with taking up physical activity was also evaluated. Patients who adhered to physical activity recommendations had significantly better quality of life. However, we were not able to establish the causal relationship between those parameters. Other authors clearly demonstrated that exercising improved quality of life in hemodialysis patients, particularly in the following domains: physical fitness, pain sensation, overall health, vitality, and mental health [4,92,93,94].

This study had several limitations. First, a relatively small group of individuals was studied. Second, the assessment of adherence to physical activity recommendations was performed using indirect methods. The fact that the study included patients from the same center can, on the one hand, be treated as a limitation. On the other hand, however, it allowed the elimination of several confounding factors, including various standards and procedures. Finally, the study was not randomized. Direct contact of the same professionally trained researcher with each participant was the strength of our study. This allowed the implementation of the same research methodology. The way of conducting the study facilitating the therapeutic education of patients regarding the benefits of physical exercise was added value.

## 5. Conclusions

Adherence to recommendations on physical activity was affected by motivation, lower levels of trait anxiety, and better quality of life, especially in the domains of sleep and physical performance. Adherence was most limited by frailty, dialysis catheter insertion, low functional fitness, diabetes as the primary cause of ESRD, extended duration of renal replacement therapy, and the female gender. Age modified the effect of recommendation awareness and disease acceptance on adherence to physical activity recommendations.

## Figures and Tables

**Table 1:** Basic sociodemographic and clinical characteristics of patients participating in the study.

Variables	Study Group (n=72)	
Age (years)	57.8±16.0	range = 19–87
Age ≥ 60, *n* (%)	38 (52.7)	
Sex, *n* (%) female/male	36 (50)/36 (50)	
Physical activity, *n* (%) yes/no	36 (50)/36 (50)	
Dialysis vintage (months)	46.2±41.4	range = 2–269
Dialysis adequacy ($Kt/V_{\mathrm{Daugirdas}}$)	1.57±0.23	range = 1.06–2.19
BMI (kg/m^2^)	24.5±4.5	range = 16.4–36.3
Weight (kg)	69.0±15.1	range = 42.5–11.5
Vascular access, *n* (%)		
arteriovenous fistulas	41 (56.9)	
arteriovenous grafts	8 (11.1)	
central venous catheters	23 (32)	
Cause of end-stage renal disease, *n* (%)		
glomerulonephritis	16 (22.2)	
diabetic renal disease	21 (29.2)	
hypertension nephropathy	12 (16.7)	
other	23 (31.9)	
CCI (points)	4.9±2.4	range = 2–12
Marital status, *n* (%) married/single	53 (73.6)/19 (26.4)	
Education, *n* (%)		
basic	10 (13.9)	
vocational training	30 (41.7)	
general	28 (38.9)	
higher	4 (5.6)	
Professional activity, *n* (%)		
work	7 (9.7)	
retirement	24 (33.3)	
disability pension	32 (44.4)	
unemployment	9 (12.5)	
Robust, *n* (%)	32 (44.4)	
Pre-frailty, *n* (%)	11 (15.3)	
Frailty, *n* (%)	29 (40.3)	
Hemoglobin (g/dL)	10.6±1.5	range = 7.7–17.5
WBC (×10^3^/µL)	6.8±1.9	range = 3.3–12.4
Creatinine (mg/dL)	7.8±2.3	range = 1.51–12.8
Sodium (mmol/dL)	138.3±2.2	range = 133.5–145.4
Potassium (mmol/dL)	5.3±0.6	range = 3.8–6.5
Urea, predialysis (mmol/L)	20.4±5.8	range = 6.9–31.5
Urea, postdialysis (mmol/L)	5.7±2.2	range = 1.4–12.7

Note: Results are the mean ± standard deviation (SD) or numbers (and percentages); significant at *p*-value < 0.05. Abbreviations: BMI, body mass index; CCI, Charlson Comorbidity Index; WBC, white blood cell.

**Table 2:** Characteristics of patients in relation to self-reported adherence to physical activity recommendations.

Variables	Activity Recommendation		*p*-Value Group A vs. NA
	A Group *n* = 36	NA Group *n* = 36	
**Age**			
≥60 years (n=38)	12 (31.6)	26 (68.4)	<0.001
<60 years (n=34)	24 (70.6)	10 (29.4)	
Cramér’s V	0.362		
**Gender**			
Women (n=36)	13 (36.1)	23 (63.9)	0.018
Men (n=36)	23 (63.9)	13 (36.1)	
Cramér’s V	0.278		
**Marital status**			
Married (n=53)	28 (52.8)	25 (47.2)	0.090
Single (n=19)	8 (42.1)	11 (57.9)	
Cramér’s V	0.420		
**Education**			
Basic (n=10)	2 (20)	8(80)	0.160
Vocational training (n=30)	15 (50)	15 (50)	
General (n=28)	17 (60.7)	11(39.3)	
Higher (n=4)	2(50)	2(50)	
Cramér’s V	0.260		
**Professional activity**			
Work (n=4)	4 (100)	0	0.004
Retirement (n=26)	8 (30.8)	18 (69.2)	
Disability pension (n=31)	20 (64.5)	11 (35.5)	
Unemployment (n=11)	4 (36.4)	7 (63.6)	
Cramér’s V	0.395		
**Vascular access**			
Arteriovenous fistulas (n=41)	26 (63.4)	15 (36.6)	0.043
Arteriovenous grafts (n=8)	2 (25)	6 (75)	
Central venous catheters (n=23)	8 (34.8)	15 (65.2)	
Cramér’s V	0.325		
**Cause of end-stage renal disease**			
Diabetic renal disease (n=21)	6 (28.6)	15 (71.4)	0.020
Hypertension nephropathy (n=12)	9 (75)	3 (25)	
Glomerulonephritis (n=16)	11 (68.8)	5 (31.2)	
Other (n=23)	10 (43.5)	13 (56.5)	
Cramér’s V	0.363		
**Mobility**			
Travel beyond place of residence (n=29)	24 (82.8)	5 (17.2)	<0.001
Place of residence (n=7)	4 (57.1)	3 (42.9)	
Walk in local area (n=5)	2 (40)	3 (60)	
Walk around the house/apartment block (n=13)	5 (38.5)	8 (61.5)	
House or apartment (n=10)	1 (10)	9 (90)	
Room (n=8)	0	8 (100)	
Cramér’s V	0.622		
**Level of visual impairment**			
Normal functioning possible (n=49)	32 (65.3)	17 (34.7)	<0.001
Slightly impaired functioning (n=14)	2 (4.3)	12 (85.7)	
Moderately impaired functioning (n=4)	0	4 (100)	
Severely impaired functioning (n=5)	2 (40)	3 (60)	
Cramér’s V	0.470		
**Evaluation of frailty**			
Robust (n=32)	24 (75)	8 (25)	<0.001
Pre-frailty (n=11)	6 (54.5)	5 (45.5)	
Frailty (n=29)	6 (20.7)	23 (79.3)	
Cramér’s V	0.500		
**Awareness of recommendations**			
Yes (n=50)	30 (60)	20 (40)	0.009
No (n=22)	6 (27.3)	16 (72.7)	
Cramér’s V	0.321		

Notes: Results are numbers (and percentages); significant at *p*-value < 0.05 for the relationship between age, gender, marital status, education, professional activity vascular access, cause of end-stage renal disease, mobility, level of visual impairment, evaluation of frailty, awareness of recommendations, and adherence; Cramér’s V is a measure of association between nominal variables, giving a value between 0 and 1. The maximum likelihood test (L_2_) was used. Abbreviations: A, adherers; NA, non-adherers.

**Table 3:** Results for factors associated with adherence to physical activity recommendations (n=72).

Variables	A Group (n=36)	NA Group (n=36)	*p*-Value (Group A vs. NA)
Age (years)	50.8±15.9	64.7±13.0	<0.001
Dialysis vintage (months)	42.5±53.2	49.9±49.5	0.099
BMI (kg/m^2^)	24.4±4.5	24.6±5.0	0.714
Weight (kg)	70.6±15.3	67.1±14.9	0.364
CCI (points)	3.9±2.0	5.9±2.6	<0.001
ADL (points)	5.9±0.4	5.0±1.9	<0.001
IADL (points)	23.6±4.0	17.1±6.6	<0.001
Barthel (points)	19.6±1.1	16.7±3.9	<0.001
AIS (points)	23.3±7.9	19.8±8.3	0.039
STAI-x1 (points)	41.9±12.6	38.8±9.4	0.233
STAI-x2 (points)	40.0±8.2	46.0±10.5	0.021
Motivation (points)	65.9±8.8	58.6±8.1	<0.001
CSHA-CFS (points)	3.6±1.0	5.1±1.5	<0.001

Note: Results are mean ± SD; significant at *p*-value < 0.05. Abbreviations: ADL, activities of daily living; IADL, instrumental ADL; AIS, acceptance of illness scale; STAI, state-trait anxiety inventory; CSHA-CFS, Canadian Study of Health and Aging Clinical Frailty Scale.

**Table 4:** Quality of life in relation to adherence to physical activity recommendations.

Scales		A Group *n* = 36	NA Group *n* = 36	*p*-Value Group A vs. NA
**Part I**	**Kidney disease related life quality domains**			
1	Symptom/problem list	77.20±13.78	70.20±14.55	0.047
2	Effects of kidney disease	65.02±18.58	61.37±18.22	0.418
3	Burden of kidney disease	46.88±28.77	37.15±28.49	0.153
4	Work status	20.83±31.22	22.22±29.01	0.851
5	Cognitive function	76.67±24.31	65.93±24.13	0.059
6	Quality of social interaction	78.15±19.52	71.48±20.17	0.161
7	Sexual function	77.31±32.70	58.93±33.34	0.092
8	Sleep	57.22±19.49	44.38±20.26	0.006
9	Social support	80.09±21.39	68.06±20.89	0.018
10	Dialysis staff encouragement	72.22±27.76	57.64±27.67	0.015
**Part II**	**General health perception scores**			
1	Physical functioning	65.97±29.67	32.78±29.37	<0.001
2	Role limitations—physical	50.00±43.77	28.47±43.18	0.042
3	Pain	58.54±34.79	47.99±35.20	0.202
4	General health	42.22±20.79	34.31±20.28	0.105
5	Emotional well-being	65.00±20.61	56.44±20.79	0.085
6	Role limitations—emotional	64.81±46.61	49.07±46.95	0.159
7	Social function	65.28±31.75	46.18±31.25	0.009
8	Energy/fatigue	52.64±22.34	36.25±21.98	0.002
9	Overall health rating	53.61±17.19	45.56±16.65	0.077
10	Patient satisfaction	63.89±19.30	59.72±19.05	0.532
11	SF-12 Physical Health Composite	39.34±11.70	32.34±11.57	<0.001
12	SF-12 Mental Health Composite	47.31±12.22	42.52±12.21	0.119

Notes: Results are the points mean ± SD; significant at *p*-value < 0.05. Evaluation was performed using the KDQOL-SF 1.3 questionnaire. Abbreviations: KDQOL-SF, kidney disease quality of life.

**Table 5:** Multivariate logistic regression analysis—factors significantly associated with adherence to physical activity recommendations.

Variables	OR	*p*-Value	95% CI
**Model A (all patients included)**			
$\chi^{2}$ 46.9; Nagelkerke $R^{2}$ 0.64; p<0.001			
CSHA-CFS (points)	0.38	0.001	0.206–0.688
Motivation (points)	1.13	0.009	1.032–1.245
Duration of hemodialysis (months)	0.99	0.047	0.972–0.999
Awareness of recommendations (1—aware; 0—unaware)	0.08	0.005	0.013–0.464
Vascular access (1—arteriovenous fistulas; 0—other)	0.09	0.007	0.017–0.530
**Model B (patients ≥60 yrs included)**			
$\chi^{2}$ 46.9; Nagelkerke $R^{2}$ 0.67; p<0.001			
CSHA-CFS (points)	0.25	0.028	0.071–0.861
Motivation (points)	1.19	0.051	1.032–1.245
AIS (points)	0.79	0.036	0.640–0.990
Duration of hemodialysis (months)	0.95	0.025	0.918–0.999
Vascular access (1—arteriovenous fistulas; 0—other)	0.09	0.045	0.008–0.950
**Model C (patients <60 yrs included)**			
$\chi^{2}$ 22.7; Nagelkerke $R^{2}$ 0.69; p<0.001			
CSHA-CFS (points)	0.13	0.012	0.026–0.633
AIS (points)	1.26	0.058	0.991–1.603
Aware of recommendations (1—aware; 0—unaware)	0.02	0.012	0.001–0.427

Notes: Odds ratio is shown with 95% CI for significant covariates. Variables that yielded *p*-values of 0.1 or lower in the initial univariate logistic regression analysis of factors were predictive of falls. Logit modeled probability that adherence = yes. Abbreviations: OR, odds ratio; CI, confidence interval.
